# Defining weaning age of camel calves in Eastern Ethiopia

**DOI:** 10.1186/2193-1801-3-313

**Published:** 2014-06-25

**Authors:** Merga B Chibsa, Yesihak Y Mummed, Mohamed Y Kurtu, Mengistu U Leta

**Affiliations:** School of Animal & Range Sciences, College of Agriculture and Environmental Sciences, Haramaya University, Post Box 138, Haramaya, Ethiopia; Department of Animal and Wildlife Sciences, University of Pretoria, Private bag X20, Hatfield, Pretoria, 0028 South Africa

**Keywords:** Camel calves, Concentrate supplementation, Weaning age

## Abstract

This experiment was conducted with the aim to define the weaning age of camel calves managed with pastoral farmers in eastern Ethiopia. Twenty camel calves (11 males and 9 females) were randomly assigned into five blocks based on their birth date. Calves within a block were further assigned to one of the four Treatments (T1, T2, T3, and T4). Calves in T_1_, T_2,_ and T_3_ were weaned at 6, 8, and 10 months of age and supplemented with concentrate from weaning up to 12 months of age, respectively. They were supplemented with a mixture of noug seed (*Guizotia abyssinica)* cake and wheat bran at a ratio of 40% and 60%, respectively. Calves in T_4_ (control) were weaned at 12 months of age, hence were not supplemented with concentrate. Calves in all treatment groups browsed natural vegetation for 8 hours a day. Post weaning performance was evaluated for all calves at 14 months of age. The mean daily concentrate intake was significantly higher (P < 0.001) in the dry season compared to the wet season. Daily weight gain was significantly (P < 0.001) affected by treatment, sex of calves, and season of birth. Calves supplemented with concentrate gained relatively more weight (P < 0.001) than calves not supplemented. Calves born during the short rainy season gained more weight than those born during the short and long dry season. Three calves died, two from T3 and one from T4. From the study it was concluded that weaning calves at 8 months of age and supplementing with concentrate to the age of 12 months of age resulted in good post weaning growth rate and survivability of calves.

## Background

The dromedary Camel (*Camelus dromedarius*) like any other herbivore animals grazing in arid rangelands are seasonally challenged with shortage of feed and scarcity of water, both in quantity and quality (Farid et al. 2010). However, they are known for their ability to survive and produce milk during dry and drought periods (Bekele et al. 2011). The foundation of a Camel herd is the calf. Calves form replacement stock without which the herd cannot grow and neither would milk be available for the Camel keepers. However, rearing of Camel calves under traditional pastoral production systems is faced with several challenges that result in high death rates of the calves. The main cause of calf mortality in African camels is supposed to be malnutrition because of competition between calf and pastoralists for milk (Yesihak and Bekele 2004). The competition become severe in mid to late stage of lactation as the dam reduce its milk and the calves become big enough to suckle more milk from the dam. A study has reported the possibility of weaning camel calves as early as 7 months of age without affecting the future performance of the animal (Aboul-Ela 1994). Calves weaned earlier than 7 months of age reached age at first service and calving at 42 and 55 months of age, respectively in the United Arab Emirates (Aboul-Ela 1994). Moreover, the possibility of milking camels in the mid to late stage of lactation by letting their calves stand in front of their dams was observed successful with some pastoral camel herders. Dams which lost their calves can also be milked by putting a dole calf made up of the skin of the deceased calf (Wilson 1998). Calves older than six months of age have the ability to browse and consume supplemental feed. These experiences indicate a possibility of weaning calves at earlier age without affecting the performance of the dam in milk production and future performance of the calves. However, identifying the exact age at which calves should be weaned was not studied earlier in Ethiopia. Therefore, this study was conducted with the aim to define the weaning age of camel calves kept by farmers in eastern Ethiopia.

## Results and discussions

### Chemical composition of the supplement

DM (dry matter), CP (crude protein), NDF (neutral detergent fiber), ADF (Acid detergent fiber), ADL (Acid detergent lignin), OM (organic matter) and Ash of the concentrate mix were 89.34, 27.80, 45.36, 23.55, 4.43, 88.96 and 8.34%, respectively.

### Nutrient acquired from supplemental concentrate

The total CP intake estimated from the total concentrate DM (Kg/day) intake was 0.44 kg/day which ranged from 431 to 450g/day (Table 1). The result was higher than 195–244 g DCP reported (Anonymous 1990) and the daily maintenance requirements of dromedary camel (100 to 150 g). This indicated that the supplemented calves had higher CP intake than their maintenance requirements.

**Table 1:** **Nutrient acquired by calves from concentrate supplemented**

Parameters	T _1_	T _2_	T _3_	Mean ± SE
Conc. DMI (kg/day)	1.61	1.62	1.55	1.59 ± 0.03
Conc. DMI (g/kg W^0.75^)	42.22	41.68	38.22	40.71 ± 2.12
Conc. DMI (g/kg BW)	12.55	12.29	11.13	11.99 ± 0.62
OMI (kg/day)	1.43	1.44	1.38	1.42 ± 0.03
OMI (g/kg W^0.75^)	37.56	37.07	34.00	36.21 ±1.57
OMI (g/kg BW)	11.16	10.93	9.90	10.66 ± 0.55
Ash intake (kg/day)	0.13	0.13	0.13	0.13 ± 2.62
Ash intake (g/kgW^0.75^)	3.52	3.48	3.19	3.40 ± 0.15
Ash intake (g/kg BW)	1.05	1.02	0.93	1.00 ± 0.05
CPI intake (kg/day)	0.45	0.45	0.43	0.44 ± 0.01
CPI (g/kg W^0.75^)	11.77	11.55	10.65	11.32 ± 0.48
CPI (g/kg BW)	3.49	3.41	3.10	3.33 ± 0.17
NDF intake (kg/day)	0.73	0.73	0.70	0.72 ± 0.01
NDF intake (g/kg W^0.75^)	19.21	18.87	17.37	18.48 ± 0.80
NDF intake (g/kg BW)	5.71	5.57	5.06	5.45 ± 0.28
ADF intake (kg/day)	0.38	0.38	0.36	0.37 ± 0.01
ADF intake (g/kg W^0.75^)	42.31	41.60	38.29	40.73 ± 1.75
ADF intake (g/kg BW)	2.96	2.89	2.63	2.83 ± 0.17
ADL intake (kg/day)	0.07	0.07	0.07	0.07 ± 0.01
ADL intake (g/kg W^0.75^)	42.31	41.60	38.31	40.74 ± 1.74
ADL intake (g/kg BW)	0.55	0. 55	0.50	0.53 ± 0.02

W^0.75^, metabolic body weight; Conc. DMI (g/kg BW), Concentrate dry matter intake g/kg body weight; OMI, Organic matter intake; CPI, Crude protein intake; NDFI, Neutral detergent fiber intake; ADF, Acid detergent fiber; ADL, Acid detergent lignin; SEM, Standard error of mean; Conc.DMI, Concentrate dry matter intake; conc. DMI (g/kgW^.75^), Concentrate dry matter intake g/kg metabolic body weight; T_1_ weaned at 6 months old; T_2_ weaned at 8 months old; T_3_ weaned at 10 months old.

### Concentrate intake by camel calves

The average concentrate consumption of camel calves was 1.59 kg DM per day. The intake in the present study was comparable to the report by Kadim et al. (2013) which was 1.6–2.25 kg DM/100 kg body weight. Concentrate intake was significantly (P < 0.001) affected by experimental season. Calves consumed more concentrate (P < 0.001) in the dry than in the wet season (Table 2). The reason for relatively higher amount of concentrate consumption in the dry season might be due to the scarcity and low quality of the forage available from the rangeland. Chimsa et al. (2013) reported that the crude protein content of mixed plant species preferred by calves in dry season was relatively lower than the content in wet season. Moreover, calves relatively spent less time browsing during dry season compared to wet season, which means less total dry matter intake from browsing/grazing thus leading to higher consumption of the concentrate. The result may justify supplementation of calves during the dry but not during the wet season.

**Table 2:** **Least Squares Means concentrate intake (kg/day) of camel calves**

Variables	Mean	SE
Overall mean	1.59	0.03
Treatment	ns	
T_1_	1.61	0.02
T_2_	1.62	0.02
T_3_	1.55	0.03
Sex	ns	
Male	1.53	0.02
Female	1.66	0.02
Experimental season	**	
Dry	1.83^a^	0.03
Wet	1.36^b^	0.04

Means with different letters (^a, b^) within a column under the same factor are different at indicated P value, ** = P< 0.001; N = number of observation; ns = non significant.

### Average daily weight gain of calves

Average daily weight gains of the calves were 0.300 kg/day. Weight gain was significantly (P < 0.001) affected by treatment, season of birth and sex of calves. Daily weight gain of calves in T_4_ (0.222 ± 0.01 kg) was relatively lower than calves in T_1_ (0.321 ± 0.01 kg), T_2_ (0.338 ± 0.01) and T_3_ (0.306 ± 0.01 kg; Table 3). The poor performance of calves in T4 (control) compared to other treatment could be due to absence of supplement and deficiency of required nutrients in forage browsed (Chimsa et al. 2013) and milk suckled (Yesihak and Bekele 2004). The weight gain of calves in T4 (control) in the current study was relatively lower than 351.6 kg/day reported for traditionally managed camels in Sudan (Bakheit et al. 2012). Moreover, the growth rate of supplemented calves was also relatively lower than the growth rate reported for Sudanese camel calves kept under semi-intensive management which was 542.3 kg/day (Bakheit et al. 2012). The difference in weight gain might be due to difference in genetic and environmental factors. Supplemented calves (T1, T2 and T3) had 27-34% higher growth rate compared to calves not supplemented (T4). This is an attribute of the supplement which supplies additional energy and protein that supports weight gain beyond the requirement for maintenance. The CP content of the supplement feed can be considered as a good source of protein since it contained over 250 g CP per kg DM (Chesworth 1992). Calves weaned at 6, 8 and 10 months of age and supplemented to the age of 12 months consumed relatively equal amount of supplement per day and yielded similar amount of gain per day. To be economically viable, supplementation should be for relatively short period of time. This will make weaning calves at 8 and 10 months of age and supplementing to the age of 12 months of age two better alternatives to wean the calves compared to weaning calves at 6 months and supplementing to the age of 12 months.

**Table 3:** **Means daily weight gain of camel calves**

Factors	Daily weight gain (kg)	
	Mean	SE
Overall mean	0.300	0.01
Treatment	**	
T_1_	0.324^a^	0.01
T_2_	0.338^a^	0.01
T_3_	0.306^a^	0.02
T_4_	0.222^b^	0.01
Season birth	**	
Long dry	0.263^b^	0.01
Short dry	0.294^b^	0.01
Short rainy	0.335^a^	0.01
Sex	**	
Female	0.330^a^	0.01
Male	0.265^b^	0.01

Means with different letters (^a, b, c^) within a column under the same factor are different at indicated P value, ** = P < 0.001; N = number of observation; DWG = daily weight gain.

Calves born during the short rainy season (0.330 ± 0.01 kg/day) had significantly (P < 0.001) higher daily weight gain compared to those born in short dry (0.294 ± 0.01) and long dry (0.265 ± 0.01) seasons. Relatively faster growth rate in short rainy season was due to the availability of forage in quantity and quality (Chimsa et al. 2013). Daily weight gain of female calves (0.330 ± 0.01) was relatively higher (P < 0.00) than male calves 265 ± 0.01) of similar age group. This is in agreement with that reported for Indian camels up to the age of 9 months (Wilson 1998). At early age females grow faster than male animals.

Three calves were died during the experimental period. Two dead calves belonged to T3 (10%) and one calf dead belong toT4 (5%). One calf that died from T3 was in the beginning of the experiment. The second calf that died from T3 and the calf from T4 died after staying in the experiment for more than ¾ of period assigned for all calves. Similar preventive and control measures were taken against disease for all calves in all treatments. Moreover, the egg per gram of feaces and packed cell volume of calves in the four treatments were not significantly different (Table 4). However, the EPG count indicated that all calves in all treatment were infested highly (EPG > 1500). This may show that calves supplemented for short period (T3) and not supplemented at all (T4) were not acquired enough resistance against gastro intestinal parasite infestation and was suggested as one of the reason for the death of calves. Effects of supplementation for different period of time on the ability to resist disease in camel calves were discussed in detail in Mohamed et al. (2013). The less chance of survivability of calves weaned at 10 months of age suggested that weaning calves at 8 months and supplementing to 12 months of age as the best alternate weaning age of camel calves.

**Table 4:** **Mortality, mean faecal egg count, larvae count and PCV of camel calves**

Treatment	Mortality (%)	Mean EPG		Mean % PCV (Range)
		Number	Transformed mean EPG (mean+S.D)	
			ns	ns
T_1_	0	2612	2.29 ±0.37	22 (18–24)
T_2_	0	1526	2.21 ± 0.35	21 (18–28)
T_3_	10	1913	2.68 ± 0.36	21 (16–26)
T_4_	5	1200	1.81 ± 0.35	20 (16–24)

EPG= egg per gram of faeces, ns= non significant.

## Conclusions

Weaning calves at early age and supplementing concentrate yield better growth rate compare to the traditional practice of weaning at 12 months of age with out supplementing. However, weaning calves at 6 months and supplementing to 12 months of age may not be economical as it yielded comparable weight gain with calves weaned at 8 and 10 months of age and supplemented to 12 months of age. Moreover, weaning calves at 10 months and supplementing to 12 months of age was too short to let calves develop resistance against diseases. It is therefore recommended that weaning calves at 8 months of age and supplemented to 12 months of age yield optimum growth rate and increase survivability of calves.

## Methods

### Description of study area

The study was conducted at Camel Research Station of Haramaya University, Ethiopia. The area is located between 9° 14’N latitude and 42° 14’E longitude at an altitude of 1300 – 1600 m above sea level. The climate condition is semi-arid with annual average rainfalls of 400 to 500 mm and average temperature of 17°C to 31°C. Seasons of the year are classified into long rainy (July–September), short rainy (March–April), long dry (October–February) and short dry (May–June) season. The soils type is sandy-dry-loam with some alluvial nature in some areas. The vegetation cover mainly includes dwarf shrubs (such as *Indigofera* species), large shrubs and trees (such as *Acacia* and *Boscia* species) and highly populated cacti.

### Study animals

Twenty Camel calves of both sexes (11 males and 9 females) between 6 and 12 months of age were used for the study. The age of the calves were obtained from the record sheet at the camel research station. During day time, all calves browsed on the communal rangeland. They browsed for about 8 hours a day (9:30 am-5:30 pm) in both the dry and wet seasons. They were provided with water once a day. During the night, they were kept in night enclosure.

### Data collection

Body weights were recorded for all camel calves in the experiment once in a week from August 2008 to August 2009. Data on the weight of calves was collected from weaning to 14 months of age for all calves.

Digital weighing scale was used to measure weight of calves. Daily weight gain was calculated by dividing difference between weekly weights by seven. Daily concentrate offered and refusals for each calf were measured and recorded. Differences in weight between concentrate offered and refused were used to calculate concentrate intake.

### Experimental design

Randomized complete block design with 4 treatments and 5 replications (animals) per treatment were used in the experiment. Twenty camel calves (11 males and 9 females) of both sexes were randomly assigned into 5 blocks based on their birth date. Calves in the block were randomly allocated to one of the 4 treatments (T_1_, T_2_, T_3,_ and T_4_). Calves in T_1_, T_2_, T_3_, and T_4_ weaned at 6, 8, 10, and 12 months of age, respectively. Calves weaned at 12 months of age were used as a control simulating practices followed by the farmers in the area. Calves in T_1_, T_2_, and T_3_ were supplemented after weaning from 6–12, 8–12, and 10–12 month of age, respectively, whereas calves in T_4_ (control) depend on browsing natural vegetation and suckled milk until 12 month of age. Camel calves that joined the respective treatments were weaned individually based on their birth date. Supplementation was done two times a day, morning (8:00–9:00am) and evening (5:00–6:00) in the individual feeding pen. The supplemented calves were given concentrate at 1.6 kg DM/100 kg live weight (Rechard 1989) with a mixture of noug seed cake and wheat bran at a ratio of 40% and 60%, respectively. Feed provisions were adjusted every fortnight based on calf body weight.

### Chemical analyses of concentrate samples

Sample of mixed concentrate offered and refused was analyzed for DM, ash, and CP (AOAC 2000). The neutral detergent fiber (NDF), acid detergent fiber (ADF), and acid detergent lignin (ADL) were analyzed (Van Soest et al. 1991).

### Statistical analysis

The data were analyzed using GLM of Minitab (1998) software. Factors showing significant difference at probability level of p < 0.05 were compared using the procedure of Tukey pairwise comparison. The statistical model used for analysis of data of concentrate consumption was:


Where, Y_ijk_ = Observation of average daily weight gain.

μ = Over all mean.

T_i_ = Fixed effect of treatment.

E_j_ = Fixed effect of season of experiment.

S_k_ = Fixed effect of sex of calf.

e_ijk_ = Effects of random error for average daily weight gain.

The statistical model used for average daily weight gain was:


Where, Y_ijk_ = Observation of average daily weight gain.

μ = Over all mean.

T_i_ = Fixed effect of treatment.

B_j_ = Fixed effect of season of birth.

S_k_ = Fixed effect of sex of calf.

e_ijk_ = Effects of random error for average daily weight gain.

## Abbreviations

DWG: Daily weight gain
T: Treatment
EPG: Egg per gram of faeces
PCV: Packed cell volume
SD: Standard deviation
SE: Standard error.
